# Host lung gene expression patterns predict infectious etiology in a mouse model of pneumonia

**DOI:** 10.1186/1465-9921-11-101

**Published:** 2010-07-23

**Authors:** Scott E Evans, Michael J Tuvim, Jiexin Zhang, Derek T Larson, Cesar D García, Sylvia Martinez Pro, Kevin R Coombes, Burton F Dickey

**Affiliations:** 1 Department of Pulmonary Medicine, University of Texas - M. D. Anderson Cancer Center, Houston, Texas, USA; 2 Center for Lung Inflammation and Infection, Texas A&M Institute for Biosciences and Technology, Houston, Texas, USA; 3 Department of Bioinformatics and Computational Biology, University of Texas - M. D. Anderson Cancer Center, Houston, Texas, USA; 4 Tecnológico de Monterrey School of Medicine, Monterrey, Nuevo León, Mexico

## Abstract

**Background:**

Lower respiratory tract infections continue to exact unacceptable worldwide mortality, often because the infecting pathogen cannot be identified. The respiratory epithelia provide protection from pneumonias through organism-specific generation of antimicrobial products, offering potential insight into the identity of infecting pathogens. This study assesses the capacity of the host gene expression response to infection to predict the presence and identity of lower respiratory pathogens without reliance on culture data.

**Methods:**

Mice were inhalationally challenged with *S. pneumoniae*, *P. aeruginosa*, *A. fumigatus *or saline prior to whole genome gene expression microarray analysis of their pulmonary parenchyma. Characteristic gene expression patterns for each condition were identified, allowing the derivation of prediction rules for each pathogen. After confirming the predictive capacity of gene expression data in blinded challenges, a computerized algorithm was devised to predict the infectious conditions of subsequent subjects.

**Results:**

We observed robust, pathogen-specific gene expression patterns as early as 2 h after infection. Use of an algorithmic decision tree revealed 94.4% diagnostic accuracy when discerning the presence of bacterial infection. The model subsequently differentiated between bacterial pathogens with 71.4% accuracy and between non-bacterial conditions with 70.0% accuracy, both far exceeding the expected diagnostic yield of standard culture-based bronchoscopy with bronchoalveolar lavage.

**Conclusions:**

These data substantiate the specificity of the pulmonary innate immune response and support the feasibility of a gene expression-based clinical tool for pneumonia diagnosis.

## Background

Pneumonias result in substantial mortality, causing more premature death and disability worldwide than any other disease [1]. Unfortunately, while patient survival depends upon the rapid identification of infecting pathogens [2], the means for prompt and accurate diagnoses of pulmonary infections remain inadequate.

Despite widespread acceptance as the diagnostic tool of choice for unexplained pulmonary infiltrates [3-5], fiberoptic bronchoscopy with bronchoalveolar lavage (BAL) provides an unambiguous diagnosis in only 25-51% of cases [2,4,6-9]. The diagnostic utility of BAL is predicated on culturing pathogens from lavage effluent, without accounting for ongoing antibiotic therapy, non-pathogenic microbial colonization, or the technical challenge of navigating the bronchoscope into involved airways. Molecular techniques, such as antigen detection and polymerase chain reaction (PCR) testing, enhance BAL sensitivity for a subset of pathogens, but still often fail to explain infiltrates [7].

Often regarded as passive gas exchange barriers, the active responses of the lungs are critical to protection from infections. In the presence of inflammatory stimuli, the respiratory epithelia rapidly recruit inflammatory cells and undergo remarkable structural and functional changes [10-13], including the release of pathogen-specific antimicrobial products [14-16].

Even in the absence of an adaptive immune system, lower metazoans like *Drosophila melanogaster *selectively respond to different classes of microorganisms following pathogen detection with conserved pattern recognition receptors [17,18]. Similarly, stereotyped pathogen-specific host innate immune responses are also observed from human dendritic cells [19], human monocytic cells [20-24], human endothelial cells [25], murine microglial cells [26], and murine jejunal epithelial cells [27]. Based upon these multiply observed tailored responses and the inflammatory capacity of pulmonary epithelium [12,28], we hypothesized that the lungs also respond selectively to different pathogens. In order to pursue the potential to achieve superior diagnostic utility in a timely manner, we interrogated this selective response to determine the etiology of pneumonias without reliance on culture data.

## Methods

### Animals and reagents

Unless otherwise specified, reagents were obtained from Sigma (St Louis, MO). All experiments were approved by the M. D. Anderson Cancer Center Institutional Animal Care and Use Committee. Specific pathogen free BALB/c mice were purchased from Harlan (Indianapolis, IN) and used in experiments at five to eight weeks old.

### Infection Model

To achieve simultaneous exposure of large numbers of mice to respiratory pathogens, mice were placed in a nebulization chamber that was sealed except for an efflux limb that vented to a low resistance filter in a biohazard hood. An AeroMist CA-209 compressed gas nebulizer (CIS-US, Inc., Bedford, MA) was used to aerosolize pathogen suspensions, driven by 10 L/min of room air and supplemented with 5% CO_2 _to promote maximal ventilation and homogeneous exposure throughout the lungs, as we have previously described [29-32]. While it is conceivable that exposure of mice to increased inspired CO_2 _concentrations might alter gene expression, our experience supports prior reports that this promotes pathogen deposition in the lungs [33,34], and our strategy involves differential gene expression analysis where all mice are exposed to the same CO_2 _environment, thus no differential effects should be detected.

### Organisms

For bacterial pathogens, the inocula were targeted to an LD_75 _by 48 h after infection. After growth to log phase, *Streptococcus pneumoniae *serotype 4, and *Pseudomonas aeruginosa *strain PA103 were each suspended in phosphate buffered saline (PBS) and delivered by aerosol. A standardized nebulization of 10 ml pathogen suspension over one hour to achieve the desired lethality required concentrations of approximately 1 × 10^10 ^CFU/per ml *S. pneumoniae *and approximately 1 × 10^11 ^CFU/ml of *P. aeruginosa*, as we have previously described [29,31].

Because *Aspergillus fumigatus *is not lethal in non-immunosuppressed BALB/c mice, we delivered the maximal reproducible concentration of organisms as limited by viscosity. This dose was 1 × 10^9 ^conidia/ml, as determined using a standard hemacytometer. Conidia of strain Af293 were stored as frozen stock (1 × 10^9 ^conidia/ml) in 20% glycerol in PBS. One ml of stock was plated on yeast extract agar plates at 37°C in 5% CO_2 _for 3 days, then harvested by gentle scraping in PBS containing 0.1% Tween-20, and the suspension was filtered through 40 μm filters, centrifuged at 2,500 × *g *for 10 min, washed, resuspended in 10 ml PBS and aerosolized over 60 min, identical to the bacterial infections. To confirm both pulmonary deposition and infective capacity of the pathogen, additional mice were challenged with the same *A. fumigatus *protocol with or without prior cyclophosphamide and cortisol immunosuppression, as previously described [31].

A sham intervention group was treated with 10 ml PBS nebulized over 60 min under the same conditions used for infectious challenges.

### Cytokine response to pathogen challenge

At designated time points after infection, mice were anesthetized and their tracheas were exposed. BAL was performed and lavage effluent cytokine concentrations were determined by ELISA, as described [29,30].

### Gene expression analysis

At designated time points after infection, gene expression microarray analysis was performed on lung homogenates from mice after challenge following leukoreduction by repeated BAL and vascular perfusion with sterile PBS [31,32]. Lungs were excised and homogenized, total RNA was extracted, and amplified cRNA was hybridized to Illumina Sentrix Mouse-6 BeadChips (Illumina, Inc., San Diego, CA). All primary data were deposited at the NCBI Gene Expression Omnibus http://www.ncbi.nlm.nih.gov/geo/, accession GSE15869) consistent with MIAME standards (see Additional File 1).

### Blinded challenges

To test the predictive ability of the gene expression data, three blinded investigators (SEE, MJT, BFD) were independently challenged to identify the infectious conditions based on gene expression patterns without reliance on culture data. After identifying characteristic changes for each condition in the gene expression analysis, the investigators were provided the data from only six transcripts that were each believed to be uniquely altered by one of the potential infectious conditions. In order to identify potentially discriminating transcripts and to assign cutoff values for a diagnostic panel we used two approaches. First, after confirming that there was no overlap of signal intensity between 2 standard deviations below a differentially upregulated gene and 2 standard deviations above the next highest condition for that transcript, we assigned a cutoff value for a positive test at 1 standard deviation below the mean signal intensity for the transcript in question. As a second approach, we created receiver operating characteristic (ROC) curves for each potentially discriminating transcript, selected from the list of differentially expressed genes. Two potentially predictive genes for each of the three infections were hand-selected for the panel, and the investigators were instructed to predict the pathogen based on the prestated rules (Additional File 2). Investigators were instructed to infer that a sample was from the sham group if the values did not meet criteria for one of the infections.

### Computer Algorithm

A computer algorithm was devised to automate the prediction of infecting organisms, based on the 18 h microarray data described above. The predictive model is a decision tree, with the first branch a decision between lungs infected with a bacterial pathogen and those not infected with bacteria. The sequential decisions are between *S. pneumoniae *and *P. aeruginosa *in the bacteria branch and between *A. fumigatus *and sham in the non-bacterial branch. Transcripts with predictive power to discern between branches were identified by fitting a linear model for each transcript, then the infectious condition of each blinded sample was sequentially predicted based on the expression of 1 to 21 discrete transcripts, with each transcript "voting" for one side of the decision tree (e.g., predicting either "bacterial" or "not bacterial"). To avoid ties when using majority vote rule, only odd numbers of predictor genes were allowed (see Additional File 1).

## Results

### Infectious pneumonia model

**Figure 1:**
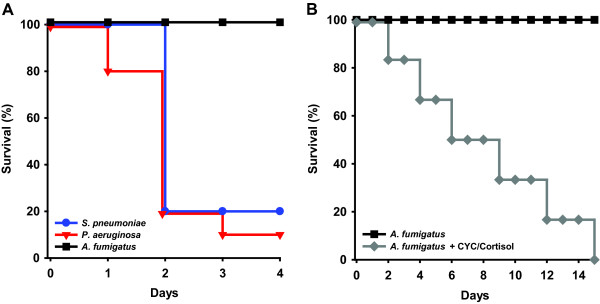
**Survival following infectious challenges**. **(A) **Using an experimental model of inhalational pneumonia in BALB/c mice, *P. aeruginosa *and *S. pneumoniae *both induced consistent mortality >80%, while mice challenged with *A. fumigatus *or PBS (sham) had 100% survival. **(B) **Mice treated with cyclophosphamide and cortisol prior to infection also consistently succumbed to *A. fumigatus *challenge, substantiating the effective delivery of pathogens to the mice (N = 10 mice/group, *p = 0.0007 vs. *A. fumigatus*, **p = 0.0001 vs. *A. fumigatus*, †p < 0.0001 vs. *A. fumigatus*).

Consistent with our prior observations [29-31], our bacterial pneumonia model yielded highly reproducible mortality (Figure 1A). No mortality was observed following fungal challenges or sham treatment. We confirmed delivery of infective conidia through the observation of highly reproducible mortality at the same inoculum for immunosuppressed mice (Figure 1B), and serial dilution culture of lung homogenates showed deposition of approximately 3 × 10^6 ^conidia per *A. fumigatus*-challenged mouse.

### Proteomic comparison

**Figure 2:**
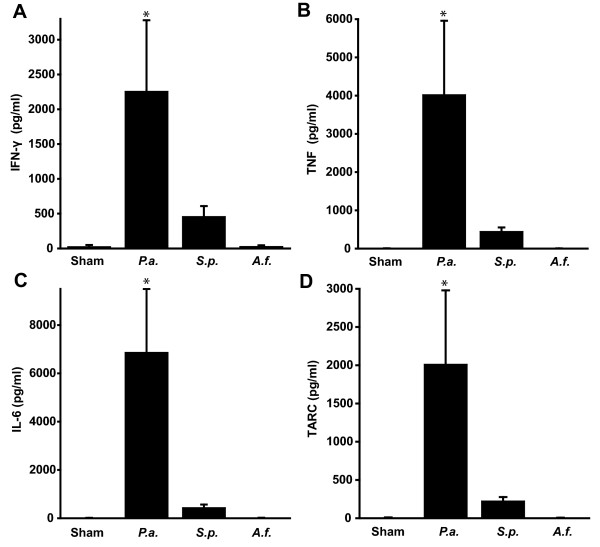
**Proteomic analysis of post-challenge BAL fluid**. Mice were challenged with aerosolized *P. aeruginosa, S. pneumoniae, A. fumigatus *or PBS (sham). 24 h later, BAL was performed and concentrations of 16 cytokines and chemokines were measured by ELISA. In all cases, *P. aeruginosa *induced the highest level of cytokine or chemokine expression, with no test identifying any other infectious condition. Shown are representative examples: **(A) **Interferon-γ, **(B) **tumor necrosis factor **(C) **Interleukin-6 and **(D) **CCL17. (mean ± SD, N = 5 mice/group, *p < 0.005 compared to all other conditions.

We initially suspected that lung cytokine responses to different pathogens might be diagnostically predictive. To test this, we compared the BAL concentration of 16 inflammatory cytokines by ELISA (Additional File 3) to determine whether this approach would be allow discernment of the conditions. Representative examples of IFN-γ, TNF-α, IL-6, and CCL-17 are shown in Figure 2 to be strongly induced by *P. aeruginosa *infection, with lesser induction by other infections. No cytokines were uniquely induced by any other pathogen.

### Infection-induced gene expression changes

**Figure 3:**
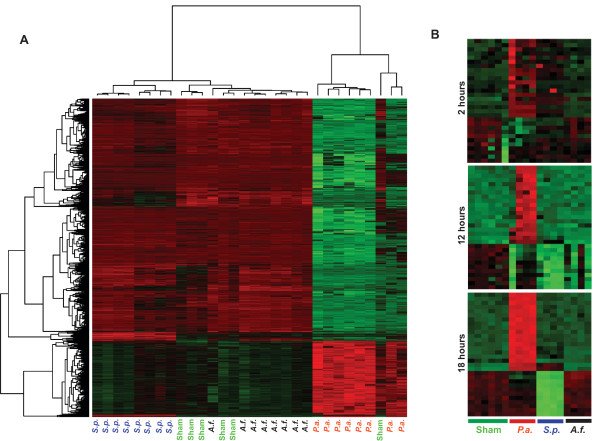
**Early development of infection-specific transcription profiles**. **(A) **Six hours after challenge with *P. aeruginosa, S. pneumoniae, A. fumigatus *or PBS (sham), lungs were removed and submitted to microarray analysis, and a heatmap was generated with green indicating decreased gene expression and red indicating increased gene expression. At this time, 4,274 genes were highly differentially expressed (FDR< 1 × 10^-7^), and by unsupervised clustering, most samples self-segregated by challenge. (N = 6 sham infected mice, 8 mice for each infection.) **(B) **The 30 genes that were most strongly differentially expressed at 18 h after infection were examined at earlier time points, demonstrating the increasing clarity of the differential pattern. (N = 6 sham infected mice, 4 mice for each infection.)

Since the protein-level cytokine response only differentiated *P. aeruginosa *from the other conditions, we interrogated the transcriptional response of differently infected lungs. Gene expression differences emerged very early after infection. Using an extremely rigorous false discovery rate (FDR) < 1 × 10^-7^, we identified 20 differentially expressed genes (DEGs) at our earliest investigated time point, 2 h after challenge. By 6 h after challenge, this number had increased to 4,274 DEGs, nearly 10% of the 45,992 oligonucleotides probed. By unsupervised clustering, the samples tended to assemble themselves into condition-specific groups even at this early time point, with grossly recognizable patterns already developing (Figure 3B).

Over the course of 12 to 18 h, the total number of DEGs at FDR of 1 × 10^-7 ^decreased to 367, but even greater condition-specific clustering was observed than at 6 h. Of these 367 DEGs, 179 were differentially expressed at both 6 h and 18 h time points. Notably, while the total number of DEGs decreased over time, the average fold-change of the remaining DEGs was generally increased.

Figure 3B demonstrates the temporal effect on gene expression in this model. The 30 most strongly differentially expressed genes at 18 h were analyzed at earlier time points, revealing progressive intensification of the gene expression patterns. Of these 30 DEGs, 18 were also differentially expressed at 6 h.

### Condition-specific transcripts

**Figure 4:**
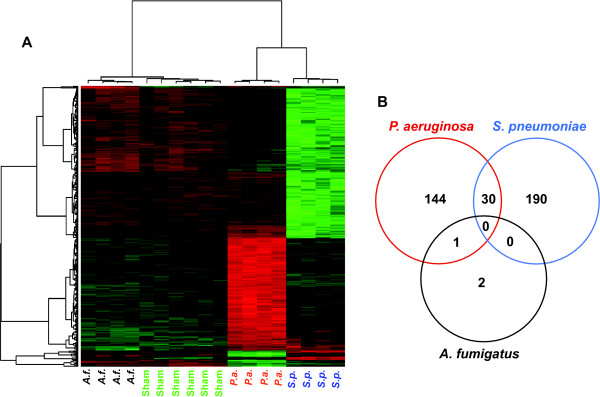
**Differential gene expression 18 hours after infectious challenge**. **(A) **A heatmap shows the expression patterns of 367 DEGs after inhalational challenge with *P. aeruginosa, S. pneumoniae, A. fumigatus *or PBS (sham). By unsupervised clustering, the samples all correctly segregate themselves by condition. **(B) **A Venn diagram indicates the striking specificity of these expression patterns, with <10% of DEGs induced or repressed by more than one condition.

By 18 h after challenge, unsupervised clustering resulted in all of the specimens correctly segregating themselves by pathogen (Figure 4A). After identifying patterns associated with each infectious condition, we focused on individual transcripts with each condition. The 367 DEGs at 18 h were sorted according to pathogen specificity (Figure 4B). Not surprisingly, the two conditions that caused mortality induced more gene expression changes than did *A. fumigatus*. However, each condition induced unique changes, and by lessening the FDR requirements, these numbers further increase.

Manual review of the 367 DEGs identified unique transcript changes for each pathogen that were included in a predictive panel. Strategies using either the magnitude of differential expression or ROC curve performance were equally efficacious for defining the prediction rule cut-off values. As shown in Additional File 4, each included transcript yielded a cutoff that achieved 100% sensitivity and 100% specificity in the 18 h training set (i.e., area under the ROC curve = 1.0). Additional Files 2 and 5 show the panel of transcripts, the prediction rules, the data provided to the blinded investigators, and their predictions. Blinded review of 18 samples at 18 h after infection resulted in 100% correct categorization of infectious conditions for all three reviewers.

When we applied these prediction rules to 18 unique samples from a validation dataset, however, the prediction accuracy dropped to only 44.4%. As shown in Additional File 6, Additional File 7 and Additional File 8, there was congruity of the blinded investigators insofar as samples were most often either correctly predicted by all three investigators or incorrectly predicted by all three investigators. No statistically significant patterns emerged among the incorrect predictions.

### Computerized prediction of infectious conditions

Since a small panel of hand selected transcripts predicted infectious conditions as well or better than traditional cultures historically perform, we sought to automate the process of prediction. We devised a multiply branching decision tree algorithm that first separated bacterial infections (*S. pneumoniae *and *P. aeruginosa*) from non-bacterial conditions (*A. fumigatus *and sham). We identified 4,799 transcripts from the training set that could distinguish these two groups. Using our predetermined criteria for predictor transcripts, we found that *Ccl4 *(chemokine C-C motif ligand 4) performed most robustly, correctly classifying all training set samples as bacteria or non-bacteria. We also found individual transcripts with very high predictive accuracy for subsequent branches of the decision tree. *Ccl3 *(chemokine C-C motif ligand 3) expression always separated *S. pneumoniae *infection from *P. aeruginosa *in the training set. A single gene, *Ttn *(titin), discriminated between *A. fumigatus *and sham in 90% of the samples, reflecting all but one sample accurately categorized by the transcript. Notably, the sham sample that was inaccurately categorized as *A. fumigatus *by *Ttn *was also predicted to be *A. fumigatus *using multiple other transcripts, and inspection of the overall gene expression profile appeared more consistent with *A. fumigatus *than sham. This raises the possibility that the mouse was inadvertently or incidentally infected with fungus. If true, the *Ttn*-based categorization would be 100% correct for this branch point, as well.

**Figure 5:**
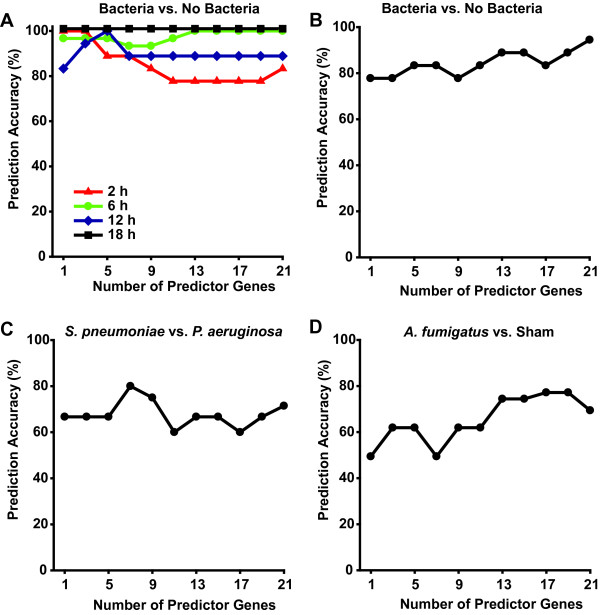
**Diagnostic accuracy of computerized gene expression interrogation**. Mice were exposed to one of four potential infectious conditions, then gene expression profiling was performed at designated time points after the challenge. **(A) **Diagnostic accuracy of algorithmic predictions of whether or not different mice were infected with bacteria, based on the time after infection and the number of transcripts used in the prediction model. **(B-D) **Rules derived from initial 18 h experiments were used to predict the infectious conditions of different mice 18 h after challenge in a separate validation set, based on number of transcripts in the algorithm. **(B) **Prediction accuracy for discriminating bacteria vs. non-bacteria. **(C) **Prediction accuracy for discriminating *S. pneumoniae *infection from *P. aeruginosa *infection. **(D) **Prediction accuracy for discriminating *A. fumigatus *from sham infection.

After identifying the most discriminant transcripts from the training set, we tested the 18 h predictor genes at other time points. Gene expression data from lungs 2 h, 6 h, and 12 h after infection were combined into a single group, then infectious predictions were made according to the algorithm. As described in Additional File 1, we used increasing odd numbers of "voting" transcripts up to 21. The prediction accuracy for discriminating bacterial infections from non-bacterial conditions was 78% for 2 h specimens, 100% for 6 h specimens, and 89% for 12 h specimens using 15 transcripts as predictors (Figure 5A). Performance of the model appeared to stabilize around 15 "voting" transcripts per branch point, with only a modest increase to 83% accuracy at 2 h using 21 predictors. There were no changes in the other time points with more than 15 predictors. The accuracy of diagnoses predicted by the second branch point was 50%, 30%, 55% and 95% for the 2, 6, 12, and 18 h specimens using 15 predictors, respectively.

The decision tree model was then tested against a unique (validation) set of gene expression data from lung homogenates collected 18 h after challenge. Using the same algorithm, the correct prediction of bacterial vs. non-bacterial status was made with 89% accuracy with 15 predictor genes and with 94.4% accuracy with 21 predictors (Figure 5B). Discrimination of *S. pneumoniae *vs. *P. aeruginosa *and of *A. fumigatus *vs. sham was achieved with >70% accuracy (Figure 5C and 5D). We again found that increasing the number of "voting" transcripts improved accuracy, with stabilization around 15 transcripts. The effect of adding additional predictor transcripts was minimal for separating the bacterial conditions from each other, but increasing from 3 to 15 transcripts correctly reclassified several samples from *A. fumigatus *to sham.

## Discussion

The informative value of host responses is increasingly recognized to differentiate between clinically confounding conditions [35]. Markers of generic inflammation have been used for decades to hint at the presence of inflammatory and infectious diseases [36,37]. More recently, host response elements have been studied to aid identification of life-threatening diseases, such as sTREM and procalcitonin in respiratory infections and sepsis [38-41]. Efforts are underway to characterize pulmonary conditions as diverse as interstitial lung diseases, pulmonary vascular diseases and asthma based on gene expression analysis [42-46]. Diagnostic host responses to *Mycobacterium tuberculosis*, are increasingly described [47-49]. Differential gene expression has been reported in the lungs following different infections [50] and gene expression profiling of leukocytes has been proposed to provide prognostic insights in the setting of lung infection [51]. However, to the best of our knowledge, this report is the first to describe a means of identifying etiologic agents of infectious pneumonia based solely on the host gene expression response.

Because of the potential ease of sampling and abundance, we first sought to discriminate between infectious conditions based on BAL cytokine levels. Using a panel of 16 cytokines, *P. aeruginosa*-infected mice were consistently differentiated from the other three conditions. This is consistent with the recent report of McConnell and colleagues who found that a panel of 18 cytokines could discriminate *P. aeruginosa*-from *S. pneumoniae*-infected mice [52]. However, while we identified a robust cytokine signature for one pathogen, we were unable to discern between the non-pseudomonal conditions by that method. Further proteomic analysis for non-cytokine host response elements may discriminate between the conditions, but our prior experience resolving low abundance peptides from BAL fluid [29] suggests that the technical challenges would offset the enhanced diagnostic capacity. Therefore, we elected to investigate host response specificity using gene expression analysis.

Our gene expression data suggest that host responses are sufficiently specific to discriminate between conditions that may be indistinguishable, such as different infectious pneumonias. While there appears to be a modest early peak of non-specific inflammation, we were surprised to identify such efficient discrimination by as early as 2 h after challenge. By 6 h after challenge, there was a robust response that waned in number of DEGs by 12 h, but clearly increased in signal amplitude of the persisting transcript changes. This durable signal increased to the 18 h time point and allowed for consistent blinded diagnoses. Remarkably, fewer than 10% of the 367 DEGs at 18 h were induced by more than one infectious condition (none by all three). Further, we found no evidence that the different infections simply induced the same gene expression patterns at different paces, rather each condition resulted in a unique gene expression profile. These findings attest to the high specificity of the host response. While the number of *Aspergillus*-regulated transcripts was low compared to the bacteria-induced DEGs, these findings are consistent with the finding of DeGregorio, et al. [53], and of Huang, et al. [19], when investigating fungus-induced gene expression in *Drosophila *and in human dendritic cells, respectively. Based on these results, we hypothesize that human lung gene expression patterns on clinical biopsy specimens will demonstrate similar specificity.

In order to systematize the otherwise subjective process of pattern-identification and to automate the process for efficiency, we devised a computerized algorithm to test whether gene expression data could predict subjects' infectious states. From a practical perspective, this strategy allowed simultaneous assessment of massive numbers of transcript permutations. More importantly, it provided diagnostic accuracy far better than that typically encountered clinically with traditional culture-based diagnostic strategies, and outperformed diagnostic predictions based on gene expression of hand selected transcripts.

The algorithm was intentionally structured as a decision tree. This allows for determination of the most relevant questions first, for sequentially increasing refinement of answers, and for the flexibility to add new branch points. In this case, based differences in available treatment options, we felt the most clinically important issue was to differentiate subjects with bacterial pneumonia from those without bacterial pneumonia. The program provided great accuracy in answering this question. The model was also robust for the secondary questions, though less so.

Typical of preliminary investigations, these data have limitations to their generalizability. Comparison of three organisms from different pathologic classes makes it impossible to know whether the effects observed are species-specific or broader effects of the group. This will be assessed in future comparisons to other members of the same classes. By design, the decision tree algorithm allows for exactly this type of modification. It is also possible that some of the gene expression changes observed in the *A. fumigatus*-infected animals may represent the effects of their immunosuppression. Given the clinical focus of our cancer center, future studies of potential drug effects will be a high priority.

Another advantage of interrogating gene expression profiles in suspected pneumonia is that it allows somewhat compartmentalized analyses of different cellular elements of the host response. Because the lungs were leukoreduced by bronchoalveolar lavage and vascular perfusion, the data presented here largely reflect responses of the epithelium. Expression patterns from simultaneously harvested alveolar macrophages will be separately analyzed and presented. Although the cellular purity is incomplete, this approach may be viewed as a preliminary model of the clinical situation where RNA can be separately obtained from epithelial cells by brushing and from alveolar macrophages by BAL. Such discrete analyses may be applied to identifying etiologies of other pulmonary condition, as well.

It could be argued that the samples were harvested sooner after initiation of infection than would be clinically possible. However, our model causes diffuse and uniform infection of the lungs, whereas clinical pneumonia generally begins with a localized infection that progresses spatially and temporally. Therefore, a clinical specimen harvested from the most recently involved lung segments will also be newly infected. Further, our observations of increasing signal intensity over time suggest that a durable diagnostic pattern will be identifiable at later stages. This will require confirmation in future, longer term studies.

## Conclusions

The early and accurate diagnosis of the etiology of pneumonia would be of great clinical benefit. These findings suggest that it may be feasible to harness the host response to inform clinicians of a patient's infectious state when pneumonia is suspected. We anticipate that this will allow for development of a clinically-relevant tool, as well as providing new insights into differences between normal and ineffective host responses to infections.

## Competing interests

The authors declare that they have no competing interests.

## Authors' contributions

SEE participated in design, performance and analysis of the infectious and microarray experiments, and wrote the manuscript. MJT participated in design, performance and analysis of the infectious experiments. JZ participated in the analysis of microarray data and writing of the manuscript. DTL participated in the performance of infectious experiments and performance microarray experiments. CDG participated analysis of microarray data. SMP participated in performance of infectious experiments. KRC participated in the analysis of microarray data and writing of the manuscript. BFD participated in design and analysis of the infectious experiments and writing of the manuscript. All authors have read and approved the final manuscript.

## Supplementary Material

Additional file 1 **Supplemental Methods**. Text file containing additional experimental and data handling methods details. Click here for file

Additional file 2 **Supplemental Table 2. Prediction rules for manually selected transcripts**. Table of rules provided to blinded investigators for predicting infectious challenges. Click here for file

Additional file 3 **Supplemental Table 1. BAL fluid cytokine levels 24 h after infection with different pathogens**. Table of BAL cytokine levels for each mouse following pathogen challenges. Click here for file

Additional file 4 **Supplemental Figure 1. Individual transcripts discriminate between infectious conditions**. Receiver operating characteristic (ROC) curves for transcripts from the 18 hour post-infection training set that discriminate *P. aeruginosa*, *S. pneumoniae*, and *A. fumigatus *from the other three potential conditions. Click here for file

Additional file 5 **Supplemental Table 3. Training set data provided to blinded investigators**. Table of gene expression data from the training set provided to blinded investigators. Click here for file

Additional file 6 **Supplemental Table 4. Validation set data provided to blinded investigators**. Table of the gene expression data from the validation set provided to blinded investigators. Click here for file

Additional file 7 **Supplemental Table 5. Condition predictions from validation set**. Table of the predictions made by each blinded investigator for each subject. Click here for file

Additional file 8 **Supplemental Table 6. Validation set performance of predictions based on hand-selected transcripts**. Table of blinded prediction performance. Click here for file
