# Supplementary material for: Host lung gene expression patterns predict infectious etiology in a mouse model of pneumonia
Source: Respir Res. 2010 Jul 23;11(1):101. doi: 10.1186/1465-9921-11-101 (PMC2914038; doi:10.1186/1465-9921-11-101)
Supplement: Additional file 1 — Supplemental Methods. Text file containing additional experimental and data handling methods details. [file 1465-9921-11-101-S1.DOC]

**SUPPLEMENTAL METHODS**

## Gene expression analysis

To evaluate lung transcriptional responses to the infectious challenges, we performed whole genome oligonucleotide gene expression microarray analysis. At designated timepoints after the infectious challenge, the mice were anesthetized and sacrificed. To reduce the gene expression signal of circulating leukocytes in the pulmonary vasculature, the mice were exsanguinated and the pulmonary arterial system was flushed with PBS. The lungs were also submitted to repeated BAL to reduce the leukocyte burden of the airspaces. The lungs were then excised, homogenized, and total RNA was isolated using the RNeasy system (Qiagen, Valencia, CA). cRNA was synthesized, then amplified, from equal masses of total RNA extracted from the lungs of infected/sham challenged mice using the Ilumina TotalPrep RNA amplification kit (Ambion, Austin, TX). Amplified cRNA was then hybridized and labeled on Sentrix Mouse-6 Expression BeadChips (Illumina, Inc., San Diego, CA). All microarrays were scanned on a BeadStation 500 (Illumina).

Data were background corrected using the robust microarray averaging (RMA) method, then transformed by taking the base-two logarithm, and quantile normalized. Analysis of the microarray output was performed using a one-way ANOVA to identify infection-induced changes. P values were modeled using a beta-uniform mixture (BUM) model [1], and combined with a false discovery rate to determine a cutoff of p values. The analysis program was written in R (R Foundation for Statistical Computing, Vienna, Austria), utilizing the Illumina library developed by Simon Lin and Pan Du, Northwestern University. Heatmaps demonstrating signal intensity display one transcript per row and one discrete lung homogenate per column, reflecting the mean signal intensity for replicates from a single mouse. No homogenates are pooled from multiple mice.

**Computer algorithm**

After multiple preliminary experiments, the training set for the algorithmic prediction rules was the original set of 18 h microarray data presented in Figure 3. Validation trials were performed using 2 h, 6 h, and 12 h microarray data from the original experiments, along with new analyses run on distinct mouse lung homogenate samples harvested at 18 h after challenge.

During the time between development of the training set and the validation set at 18 h, Illumina introduced a newer version of the Sentrix Mouse-6 Beadchip (v1.0b was replaced by v1.1). The two probe sets are largely overlapping, but a limited number of discriminate transcripts from the earlier set may not be present in the later set. In order to make the analysis consistent with previous ones, after background correction and log_2_ transformation, we performed quantile normalization such that the final quantile distribution of the validation set is the same as the quantile normalized for the 18 h data from the training set.

The predictive model is a decision tree, and the first branch is a decision between lungs infected with bacterial pathogen and those not infected with bacteria. The sequential decisions are between *S. pneumoniae* and *P. aeruginosa* in the bacteria branch and between *A. fumigatus* and sham in the non-bacterial branch. Transcripts that demonstrated discriminating ability for the given branch point in the training set were examined in a blinded manner in the validation set. We interrogated the 18,814 genes that were called present on at least three arrays in the initial 18 h dataset. Transcripts with predictive power were identified by fitting a linear (one-way ANOVA) model for each transcript and recording both the p-value from an F-test of significance and the “prediction accuracy” as defined below.

Expression values for each transcript were standardized by subtracting the mean expression of sham samples and dividing by the sham sample standard deviation. In order to predict the first branch in the decision tree, we focused on genes whose expression in bacterial samples separate completely from non-bacterial samples. The expression cutoff was defined as the midpoint between the means of the middle two of the four groups (or just the midpoint when distinguishing two groups at lower branches of the decision tree). Assuming normal distributions for each type, we calculated the lower bound of prediction accuracy as the probability of obtaining expression values beyond the cutoff value. Prediction accuracy and cutoffs were computed for each branch in the tree.

To be included in any branch of the algorithm, potential predictor transcripts had to satisfy the following criteria: (1) be significantly differentially expressed as detected by a linear model with a false discovery rate (FDR) < 0.001 in the 18 h dataset, and (2) have prediction accuracy larger than the cutoff. At 18 h after infection, there were 7,130 significant transcripts at FDR < 0.001, 165 of which had prediction accuracy >80%. These transcripts were ranked by prediction accuracy prior to inclusion in the decision tree.

Each blinded sample was sequentially predicted with expression of 1 to 21 discrete transcripts to determine the accuracy of their prediction, with each transcript “voting” for one side of the decision tree (e.g., predicting either “bacterial” or “not bacterial”). To avoid ties when using majority vote rule, we only allowed odd numbers of predictor genes.

**SUPPLEMENTARY REFERENCES**

1. Pounds S, Morris SW (2003) Estimating the occurrence of false positives and false negatives in microarray studies by approximating and partitioning the empirical distribution of p-values. Bioinformatics 19: 1236-1242.
