# Supplementary material for: Host lung gene expression patterns predict infectious etiology in a mouse model of pneumonia
Source: Respir Res. 2010 Jul 23;11(1):101. doi: 10.1186/1465-9921-11-101 (PMC2914038; doi:10.1186/1465-9921-11-101)
Supplement: Additional file 2 — Supplemental Table 2. Prediction rules for manually selected transcripts. Table of rules provided to blinded investigators for predicting infectious challenges. [file 1465-9921-11-101-S2.DOC]

**Supplemental Table 1. Prediction rules for manually selected transcripts.** Six genes were selected for use in a small panel of transcripts based on their hypothesized ability to discriminate between conditions. Three blinded investigators were instructed to use the prediction rules below to attempt to identify the infectious condition of 18 samples. If a sample did not meet criteria for any of the conditions, the investigators were instructed to infer sham infection. If no definite answer was obvious, investigators were asked to guess the most likely condition.

| **Target ID** | **Symbol** | **Definition** | **Prediction Rule (rlu)** |
| --- | --- | --- | --- |
| A = scl014825.4_307-S | Cxcl1 | Chemokine (C-X-C motif) ligand 1 | >3351 for P. aeruginosa |
| B = scl55015.6.1_13-S | Timp1 | Tissue inhibitor of metalloproteinase 1 | >3825 for P. aeruginosa |
| C = ri|1810044B19|ZX00043A03|AK007767|732-S | Cnot7 | CCR4-NOT transcription complex subunit 7 | >4930 for S. pneumoniae |
| D = ri|4930512K19|PX00033C22|AK015776|792-S | Sf3A60 | Splicing factor 3A subunit 3 (Spliceosome associated protein 61) | >1025 for S. pneumoniae |
| E = scl30620.3.1_5-S | Cox6a2 | Cytochrome c oxidase subunit VI a polypeptide 2 | >2700 for A. fumigatus |
| F = scl44295.19.197_54-S | Ryr2 | Ryanodine receptor 2 cardiac | >1024 for A. fumigatus |
