# Supplementary material for: Host lung gene expression patterns predict infectious etiology in a mouse model of pneumonia
Source: Respir Res. 2010 Jul 23;11(1):101. doi: 10.1186/1465-9921-11-101 (PMC2914038; doi:10.1186/1465-9921-11-101)
Supplement: Additional file 3 — Supplemental Table 1. BAL fluid cytokine levels 24 h after infection with different pathogens. Table of BAL cytokine levels for each mouse following pathogen challenges. [file 1465-9921-11-101-S3.DOC]

**Supplemental Table 2. BAL fluid cytokine levels 24 h after infection with different pathogens.**

|  | **GMCSF** | **IFNg** | **IL2** | **IL4** | **IL6** | **IL10** | **IL13** | **TNFa** |
| --- | --- | --- | --- | --- | --- | --- | --- | --- |
|  | pg/ml | pg/ml | pg/ml | pg/ml | pg/ml | pg/ml | pg/ml | pg/ml |
| *A. fumigatus* | 2.5 | 32.3 | 106.9 | 4.8 | 9.4 | 19.6 | 36.3 | 5.0 |
| *A. fumigatus* | 8.3 | 46.5 | 55.7 | 6.5 | 14.2 | 37.3 | 69.3 | 6.2 |
| *A. fumigatus* | 1.0 | 9.0 | 109.6 | 4.5 | 5.5 | <3.1 | 19.7 | 4.0 |
| *A. fumigatus* | 1.9 | 16.5 | 104.2 | 6.1 | 8.2 | 5.4 | 33.4 | 4.9 |
| *A. fumigatus* | 5.3 | 40.8 | 111.6 | 4.1 | 24.2 | 27.9 | 36.3 | 6.3 |
| Sham | 1.2 | 23.8 | 99.2 | 3.0 | 5.4 | 12.5 | 32.6 | 3.8 |
| Sham | <0.4 | 9.0 | 93.0 | 2.5 | 4.8 | 4.0 | 20.9 | 2.3 |
| Sham | 1.8 | 23.0 | 97.5 | 3.4 | 12.1 | 17.2 | 31.7 | 6.1 |
| Sham | 6.6 | 65.8 | 25.0 | 10.7 | 17.5 | 24.1 | 50.1 | 9.6 |
| Sham | 2.2 | 13.8 | 99.4 | 4.2 | 6.2 | 20.0 | 27.1 | 5.1 |
| *P. aeruginosa* | 62.4 | 2229.6 | 337.5 | 1158.4 | 8425.4 | 304.0 | 183.2 | 2945.0 |
| *P. aeruginosa* | 35.1 | 1741.1 | 340.5 | 888.0 | 5087.7 | 284.4 | 145.9 | 2162.2 |
| *P. aeruginosa* | 55.8 | 1084.5 | 232.3 | 574.0 | 3204.0 | 269.4 | 161.9 | 3272.1 |
| *P. aeruginosa* | 94.7 | 3838.7 | 313.6 | 1567.2 | 9441.8 | 354.4 | 298.1 | 7072.6 |
| *P. aeruginosa* | 77.8 | 2404.4 | 500.3 | 1100.1 | 8211.2 | 450.6 | 323.6 | 4681.6 |
| *S. pneumoniae* | 21.5 | 325.5 | 108.9 | 16.7 | 294.4 | 225.6 | 70.5 | 402.4 |
| *S. pneumoniae* | 14.6 | 673.1 | 106.0 | 16.5 | 332.4 | 111.1 | 40.8 | 384.7 |
| *S. pneumoniae* | 19.5 | 365.3 | 79.7 | 16.3 | 536.5 | 138.5 | 63.4 | 404.9 |
| *S. pneumoniae* | 24.7 | 380.7 | 133.4 | 17.8 | 454.8 | 148.6 | 86.2 | 433.8 |
| *S. pneumoniae* | 30.5 | 555.5 | 150.3 | 26.7 | 586.0 | 169.9 | 74.8 | 630.5 |

|  | **VEGF** | **Eotaxin** | **KC** | **JE** | **MCP5** | **MIP2** | **Rantes** | **TARC** |
| --- | --- | --- | --- | --- | --- | --- | --- | --- |
|  | pg/ml | pg/ml | pg/ml | pg/ml | pg/ml | pg/ml | pg/ml | pg/ml |
| *A. fumigatus* | 1.0 | 114.7 | 5.4 | 1.9 | 1.7 | 24.1 | 1.5 | 3.8 |
| *A. fumigatus* | 2.0 | 232.2 | 14.5 | 3.7 | 3.3 | 29.4 | 4.6 | 8.9 |
| *A. fumigatus* | 0.9 | 31.9 | 3.2 | 1.0 | 0.7 | 27.2 | <0.4 | 1.4 |
| *A. fumigatus* | 0.6 | 104.3 | 3.6 | 1.0 | 2.6 | 26.9 | 1.2 | 4.6 |
| *A. fumigatus* | 1.2 | 60.7 | 4.7 | 1.0 | 1.1 | 29.5 | 0.7 | 2.9 |
| Sham | 2.6 | 43.1 | 6.5 | 0.9 | 0.6 | 34.5 | 1.6 | 1.9 |
| Sham | 1.3 | 18.4 | 4.1 | 0.5 | 0.4 | 30.3 | <0.4 | 1.4 |
| Sham | 2.0 | 98.8 | 7.7 | 1.0 | 2.6 | 28.1 | 1.3 | 6.4 |
| Sham | 1.5 | 309.0 | 10.1 | 3.7 | 6.4 | <0.8 | 2.5 | 13.9 |
| Sham | 1.4 | 46.2 | 6.4 | 1.3 | 0.9 | 30.1 | 2.0 | 2.4 |
| *P. aeruginosa* | 31.2 | 888.1 | 693.9 | 1158.5 | 601.8 | 304.0 | 91.6 | 1472.5 |
| *P. aeruginosa* | 17.6 | 695.3 | 699.9 | 888.0 | 363.4 | 284.3 | 72.9 | 1081.1 |
| *P. aeruginosa* | 27.9 | 436.1 | 480.8 | 574.0 | 228.9 | 269.4 | 81.0 | 1636.0 |
| *P. aeruginosa* | 47.3 | 1526.9 | 645.6 | 1567.4 | 674.4 | 354.3 | 149.1 | 3536.3 |
| *P. aeruginosa* | 38.9 | 957.1 | 1021.1 | 1100.2 | 586.5 | 450.4 | 161.8 | 2340.8 |
| *S. pneumoniae* | 10.7 | 134.0 | 228.1 | 16.7 | 21.0 | 225.6 | 35.3 | 201.2 |
| *S. pneumoniae* | 7.3 | 273.1 | 222.2 | 16.5 | 23.7 | 111.1 | 20.4 | 192.3 |
| *S. pneumoniae* | 9.8 | 150.0 | 167.7 | 16.3 | 38.3 | 138.6 | 31.7 | 202.4 |
| *S. pneumoniae* | 12.3 | 156.2 | 278.8 | 17.8 | 32.5 | 148.7 | 43.1 | 216.9 |
| *S. pneumoniae* | 15.3 | 226.2 | 313.3 | 26.7 | 41.9 | 170.0 | 37.4 | 315.2 |
