# Supplementary material for: Host lung gene expression patterns predict infectious etiology in a mouse model of pneumonia
Source: Respir Res. 2010 Jul 23;11(1):101. doi: 10.1186/1465-9921-11-101 (PMC2914038; doi:10.1186/1465-9921-11-101)

**Supplemental Figure 1. Individual transcripts discriminate between infectious conditions.** Receiver operating characteristic (ROC) curves for transcripts from the 18 hour post-infection training set that discriminate **(A)** *P. aeruginosa*, **(B)** *S. pneumoniae*, and **(C)** *A. fumigatus* from the other three potential conditions. For each transcript, the cut-off value for signal intensity that achieves an area under the curve (AUC) of 1.0 is shown. (N = 4 mice/group.)


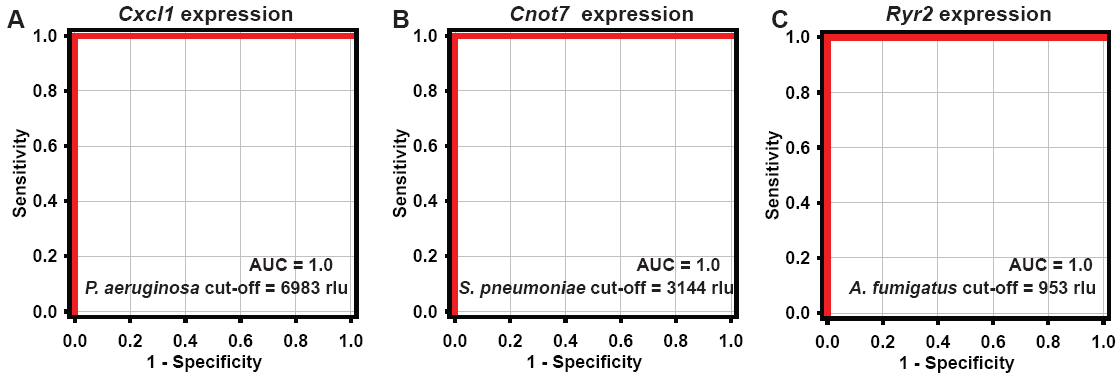

Supplement: Additional file 4 — Supplemental Figure 1. Individual transcripts discriminate between infectious conditions. Receiver operating characteristic (ROC) curves for transcripts from the 18 hour post-infection training set that discriminate P. aeruginosa, S. pneumoniae, and A. fumigatus from the other three potential conditions. [file 1465-9921-11-101-S4.DOC]
