# Supplementary material for: Host lung gene expression patterns predict infectious etiology in a mouse model of pneumonia
Source: Respir Res. 2010 Jul 23;11(1):101. doi: 10.1186/1465-9921-11-101 (PMC2914038; doi:10.1186/1465-9921-11-101)
Supplement: Additional file 5 — Supplemental Table 3. Training set data provided to blinded investigators. Table of gene expression data from the training set provided to blinded investigators. [file 1465-9921-11-101-S5.DOC]

**Supplemental Table 3. Training set data provided to blinded investigators.** Investigators were given the following data about 18 unknown samples and asked to predict the infectious condition based on the rules in Supplemental Table 2.

|  | **1** | **2** | **3** | **4** | **5** | **6** | **7** | **8** | **9** | **10** | **11** | **12** | **13** | **14** | **15** | **16** | **17** | **18** |
| --- | --- | --- | --- | --- | --- | --- | --- | --- | --- | --- | --- | --- | --- | --- | --- | --- | --- | --- |
| A | 331 | 259 | 19855 | 16038 | 18792 | 266 | 13482 | 345 | 255 | 484 | 287 | 342 | 344 | 215 | 287 | 279 | 338 | 405 |
| B | 418 | 437 | 21922 | 20554 | 25502 | 332 | 14677 | 503 | 387 | 473 | 282 | 373 | 370 | 334 | 325 | 322 | 480 | 433 |
| C | 5818 | 1266 | 308 | 304 | 311 | 280 | 186 | 237 | 262 | 329 | 254 | 234 | 5021 | 298 | 337 | 203 | 5404 | 7101 |
| D | 1110 | 862 | 360 | 438 | 470 | 695 | 259 | 527 | 735 | 603 | 600 | 465 | 1190 | 782 | 582 | 294 | 1397 | 1028 |
| E | 385 | 326 | 737 | 2108 | 566 | 2878 | 398 | 941 | 3632 | 1147 | 3376 | 1428 | 344 | 2511 | 1208 | 879 | 310 | 351 |
| F | 258 | 261 | 320 | 757 | 259 | 1164 | 198 | 465 | 1250 | 510 | 1222 | 807 | 214 | 1100 | 563 | 348 | 246 | 235 |
| Correct | Sp | Sham | Pa | Pa | Pa | Af | Pa | Sham | Af | Sham | Af | Sh | Sp | Af | Sham | Sham | Sp | Sp |
