# Supplementary material for: Host lung gene expression patterns predict infectious etiology in a mouse model of pneumonia
Source: Respir Res. 2010 Jul 23;11(1):101. doi: 10.1186/1465-9921-11-101 (PMC2914038; doi:10.1186/1465-9921-11-101)
Supplement: Additional file 6 — Supplemental Table 4. Validation set data provided to blinded investigators. Table of the gene expression data from the validation set provided to blinded investigators. [file 1465-9921-11-101-S6.DOC]

**Supplemental Table 4. Validation set data provided to blinded investigators.** Investigators were given the following data about 18 unknown samples and asked to predict the infectious condition based on the rules in Supplemental Table 2.

|  | 1 | 2 | 3 | 4 | 5 | 6 | 7 | 8 | 9 | 10 | 11 | 12 | 13 | 14 | 15 | 16 | 17 | 18 |
| --- | --- | --- | --- | --- | --- | --- | --- | --- | --- | --- | --- | --- | --- | --- | --- | --- | --- | --- |
| A | 310 | 1109 | 365 | 928 | 8767 | 359 | 340 | 196 | 570 | 5493 | 1757 | 1376 | 851 | 478 | 8262 | 332 | 214 | 26668 |
| B | 330 | 2051 | 323 | 810 | 6411 | 402 | 386 | 374 | 288 | 2694 | 1705 | 1834 | 1433 | 327 | 6339 | 492 | 981 | 874.6 |
| C | 238 | 1467 | 201 | 8195 | 153 | 325 | 170 | 8913 | 162 | 183 | 172 | 287 | 450 | 426 | 158 | 656 | 767 | 582.2 |
| D | 2050 | 4607 | 676 | 5985 | 148 | 2987 | 2385 | 7561 | 1096 | 1591 | 471 | 1876 | 688 | 1183 | 137 | 3737 | 1471 | 2794.8 |
| E | 1280 | 1157 | 1371 | 868 | 473 | 12718 | 490 | 3716 | 2944 | 7519 | 1192 | 3658 | 308 | 36541 | 497 | 9377 | 362 | 671 |
| F | 78 | 8 | 83 | 45 | 99 | 86 | 90 | -4 | 83 | 80 | 92 | 60 | -55 | 75 | 101 | 49 | 9 | -23.2 |
