# Supplementary material for: Host lung gene expression patterns predict infectious etiology in a mouse model of pneumonia
Source: Respir Res. 2010 Jul 23;11(1):101. doi: 10.1186/1465-9921-11-101 (PMC2914038; doi:10.1186/1465-9921-11-101)
Supplement: Additional file 7 — Supplemental Table 5. Condition predictions from validation set. Table of the predictions made by each blinded investigator for each subject. [file 1465-9921-11-101-S7.DOC]

**Supplemental Table 5. Condition predictions from validation set.** The blinded predictions of each observer are presented along with the actual (unblinded) condition for each of 18 unknown samples in the validation set.

|  | 1 | 2 | 3 | 4 | 5 | 6 | 7 | 8 | 9 | 10 | 11 | 12 | 13 | 14 | 15 | 16 | 17 | 18 |
| --- | --- | --- | --- | --- | --- | --- | --- | --- | --- | --- | --- | --- | --- | --- | --- | --- | --- | --- |
| Observer 1 | Sp | Sp | Sham | Sp | Pa | Af | Sp | Sp | Af | Pa | Sham | Sp | Sham | Af | Pa | Sp | Sp | Pa |
| Observer 2 | Sp | Sp | Af | Sp | Pa | Af | Sp | Sp | Af | Pa | Sham | Af | Sham | Af | Sp | Af | Sp | Pa |
| Observer 3 | Sham | Sp | Sham | Sp | Pa | Af | Sp | Sp | Af | Af | Pa | Pa | Sham | Af | Pa | Af | Sham | Pa |
| **Correct**  **Description** | **Af** | **Sp** | **Sham** | **Af** | **Pa** | **Af** | **Sham** | **Sham** | **Af** | **Sp** | **Sp** | **Sp** | **Pa** | **Sham** | **Pa** | **Af** | **Sp** | **Pa** |
| Correct  Prediction | 0/3 | 3/3 | 2/3 | 0/3 | 3/3 | 3/3 | 0/3 | 0/3 | 3/3 | 0/3 | 0/3 | 1/3 | 0/3 | 0/3 | 2/3 | 2/3 | 2/3 | 3/3 |
