# Supplementary material for: Host lung gene expression patterns predict infectious etiology in a mouse model of pneumonia
Source: Respir Res. 2010 Jul 23;11(1):101. doi: 10.1186/1465-9921-11-101 (PMC2914038; doi:10.1186/1465-9921-11-101)
Supplement: Additional file 8 — Supplemental Table 6. Validation set performance of predictions based on hand-selected transcripts. Table of blinded prediction performance. [file 1465-9921-11-101-S8.DOC]

**Supplemental Table 6.** **Validation set performance of predictions based on hand-selected transcripts.**

|  | **Correct Diagnosis** | **Incorrect Diagnosis** |
| --- | --- | --- |
| **Observer 1** | 9/18 | 9/18 |
| **Observer 2** | 7/18 | 11/18 |
| **Observer 3** | 8/18 | 10/18 |
